# Transgenerational Memory of Phenotypic Traits in Plants: Epigenetic Regulation of Growth, Hormonal Balance, and Stress Adaptation

**DOI:** 10.3390/cimb47060404

**Published:** 2025-05-29

**Authors:** Erna Karalija, Saida Ibragić, Sabina Dahija, Dunja Šamec

**Affiliations:** 1 Department of Biology, Faculty of Science, University of Sarajevo, Zmaja od Bosne 35, 71000 Sarajevo, Bosnia and Herzegovina; erna.k@pmf.unsa.ba (E.K.); sabina.dahija@pmf.unsa.ba (S.D.); 2 Department of Chemistry, Faculty of Science, University of Sarajevo, Zmaja od Bosne 33, 71000 Sarajevo, Bosnia and Herzegovina; saida.i@pmf.unsa.ba; 3 Department of Food Technology, University North, Trg Dr. Žarka Dolinara 1, 48 000 Koprivnica, Croatia

**Keywords:** crop improvement, epigenetic priming, epigenetic inheritance, hormonal regulation, plant resilience, stress memory, transgenerational adaptation

## Abstract

Plants exhibit remarkable adaptability to environmental stresses, with epigenetic modifications playing a key role in stress memory and adaptation. This review explores how epigenetic mechanisms influence hormonal regulation in plants, shaping growth, development, and stress responses. Specifically, we focus on the roles of DNA methylation, histone modifications, and small RNAs in modulating auxin, abscisic acid (ABA), gibberellin (GA), and jasmonic acid (JA) pathways. These pathways influence the plant’s ability to cope with abiotic and biotic stresses and can be inherited by progeny, enhancing stress resilience across generations. By understanding the epigenetic regulation of these hormones, we aim to provide insights into how epigenetic priming can be harnessed in crop improvement to address the challenges posed by climate change.

## 1. Introduction

Environmental stresses such as drought, salinity, extreme temperatures, and pathogen attacks have become increasingly problematic for global agricultural productivity, exacerbated by climate change. In response, plants have evolved diverse physiological, biochemical, and molecular mechanisms to cope with such stresses. One remarkable mechanism that has emerged is transgenerational stress memory, where plants “remember” past stress events and pass this memory to their progeny, enhancing their ability to adapt to recurring environmental challenges [1,2]. Plants, as sessile organisms, have evolved sophisticated mechanisms to “remember” environmental stresses, enabling them to respond more effectively upon re-exposure. This phenomenon, known as stress memory, manifests in two main forms: somatic and transgenerational memory [3,4].

Somatic stress memory refers to the retention of stress-induced epigenetic modifications—such as DNA methylation, histone modifications, and the accumulation of small RNAs—within an individual plant’s lifespan. These changes allow for a faster or more robust transcriptional response during subsequent stress events. For example, in *Arabidopsis thaliana*, drought-induced memory genes such as RD29A retain permissive chromatin states (e.g., H3K4me3) and poised RNA Polymerase II during recovery, enabling rapid re-induction upon renewed drought stress [4,5].

In contrast, transgenerational stress memory involves the inheritance of epigenetic marks that escape meiotic resetting and are transmitted to progeny. These heritable modifications include DNA methylation maintained by *MET1* or *DRM2*, histone marks such as H3K27me3, and the activity of RNA-directed DNA methylation (RdDM) pathways. Such mechanisms have been shown to confer improved stress resilience in offspring, as seen in wheat and rice exposed to drought or salt stress, where progeny exhibit altered expression of ABA- and auxin-related genes [6,7].

**Figure 1:**
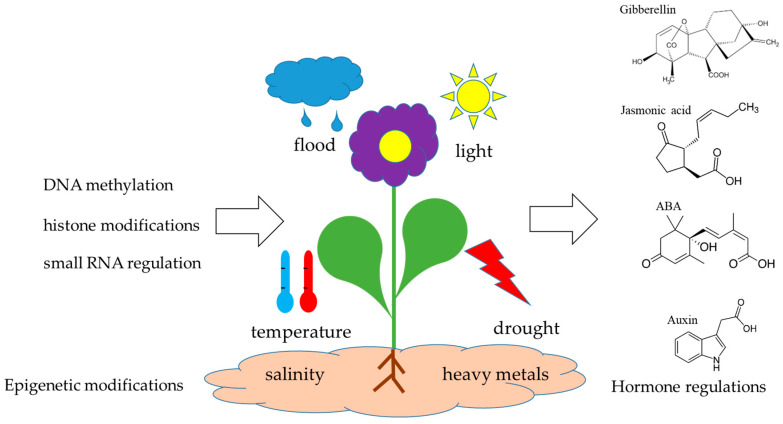
Epigenetic alterations significantly impact plant growth, development, and responses to environmental stressors, particularly through the regulation of plant hormones such as abscisic acids (ABAs), auxins, gibberellins (GAs), and jasmonic acids (JAs), which play central roles in stress signaling and resilience.

While the genetic basis of stress tolerance has been extensively studied, the role of epigenetic mechanisms in plant stress responses remains an evolving field. Epigenetic modifications, such as DNA methylation, histone modifications, and small RNA regulation, do not alter the DNA sequence but influence gene expression, thereby modifying phenotypic traits related to stress adaptation [3]. These epigenetic alterations significantly impact plant growth, development, and responses to environmental stressors, particularly through the regulation of plant hormones such as abscisic acids (ABAs), auxins, gibberellins (GAs), and jasmonic acids (JAs), which play central roles in stress signaling and resilience (Figure 1).

Despite the increasing body of research on plant stress memory, significant gaps remain in understanding the mechanisms by which epigenetic modifications regulate hormonal pathways, particularly in the context of transgenerational memory. Specifically, how these epigenetic modifications influence progeny fitness, how stable or reversible these modifications are under fluctuating conditions, and how the different epigenetic mechanisms interact to regulate hormonal responses are not well understood. Furthermore, the long-term implications of epigenetic priming for crop improvement under field conditions remain an open area for investigation.

This review aims to address these gaps by exploring how epigenetic regulation of hormonal pathways influences stress adaptation and resilience. We will explore the role of DNA methylation, histone modifications, and small RNA regulation in shaping hormonal balance in plants and examine how these modifications can be inherited, enhancing stress tolerance across generations. We will also discuss the potential applications of epigenetic priming in crop improvement, with a focus on future directions that could enhance our understanding of epigenetic memory in plants.

## 2. Epigenetic Mechanisms Governing Hormonal Regulation

Epigenetic modifications, particularly DNA methylation, have emerged as central regulatory players that govern the dynamic transcription of hormone-related genes in plants. Through changes in cytosine methylation at promoters and gene bodies, DNA methylation orchestrates hormonal biosynthesis, signaling, and downstream responses, contributing to the fine-tuning of plant growth, development, and environmental plasticity [8,9].

### 2.1. DNA Methylation Regulates Hormonal Synthesis and Signaling: A Crucial Epigenetic-Hormonal Interface in Plant Development and Stress Adaptation

DNA methylation influences hormone levels by directly targeting genes involved in their biosynthetic pathways. In soybeans, experimental demethylation via 5-azacytidine (5-azaC) significantly altered the concentrations of ABA, auxin (IAA), and ethylene, indicating that the methylation state modulates hormone biosynthesis from the early stages of seedling development [10]. The upregulation of *DRM2* and downregulation of *ROS1* in roots under hypomethylating conditions suggests methylation-dependent control of hormone-related transcriptional cascades.

In *Capsicum annuum*, ripening-associated hypomethylation was found to induce ABA biosynthesis genes while repressing auxin and gibberellin biosynthesis, suggesting a role of DNA methylation in the hormonal reprogramming that governs the ripening process [11]. The expression of *CaDML2*-*like* (a demethylase) was induced by exogenous ABA, and conversely, IAA promoted transcription of *CaMET1*-*like1*, revealing feedback between methylation machinery and hormonal signals.

Methylation of DNA impacts the transcriptional regulation of entire metabolic cascades by altering chromatin accessibility and the binding affinity of transcription factors to hormone biosynthesis genes. For instance, DNA hypomethylation enhances growth-sustaining hormonal pathways under cadmium stress in *Arabidopsis thaliana*, particularly auxin, cytokinin, and gibberellin pathways, in the methylation-defective ddc mutant [12].

Recent evidence demonstrates that plant hormones can regulate DNA methylation by altering the expression of methyltransferases and demethylases. Using GUS reporter lines in *Arabidopsis*, Bennett et al. [13] showed that phytohormones such as ABA, auxin, ethylene, SA, cytokinin, and GA dynamically alter promoter activity of key genes involved in both de novo methylation (*DRM1/2/3*) and demethylation (*ROS1, DME, DML2, DML3*). These hormone-induced shifts in the expression of methylation-related genes were confirmed by RT-qPCR, substantiating the feedback loop wherein hormonal signals influence the epigenetic landscape [13].

For example:

- Auxin decreased *DRM2* and *DML3* expression in roots and shoots, while slightly upregulating *CMT3*.
- ABA upregulated *VIM1* (a *MET1* cofactor for CG methylation) in roots and shoots but suppressed *DRM3* and *DML3*.
- GA treatment enhanced the expression of *DML2* and *DML3*, suggesting active demethylation supports GA-mediated growth.
- SA broadly suppressed *MET1*, *VIM1*, *DRM2*, and demethylases like *ROS1*, reducing methylation turnover.

The hormonal regulation of methylation genes is highly tissue- and stage-specific. For instance, in reproductive tissues, *DRM2* and *DME* are co-expressed in petals, anthers, and stigma, suggesting coordinated methylation–demethylation dynamics are essential for flower development [13]. During seed development, *DRM2*, *MET1*, and *DME* were highly active in both the endosperm and embryo, reflecting their role in shaping hormone-responsive transcription during embryogenesis [13].

Methylation profiles in root meristems and shoot apices align with the high activity of hormone signaling and biosynthesis. The activation of demethylases in mature root tissues versus methyltransferases in root tips indicates developmental reprogramming influenced by hormonal flux and epigenetic remodeling [13].

The ability of DNA methylation to modulate hormone levels enables plants to prioritize either growth or defense responses based on environmental cues. For instance, SA, typically associated with biotic stress, represses genes for both methylation and demethylation in roots and older leaves, possibly stabilizing a defense-associated gene expression state [13]. In contrast, gibberellin promotes the expression of demethylases, aligning with growth activation and stress recovery [13]. These findings suggest that DNA methylation acts not only as a molecular memory of hormonal responses but also as a regulatory integrator, ensuring a dynamic balance between plasticity and stability in plant hormone networks.

### 2.2. Histone Modifications in Hormonal Signaling: Epigenetic Regulation of Plant Development and Stress Responses

Histone modifications are pivotal chromatin-level epigenetic mechanisms that regulate transcriptional outcomes in response to developmental and environmental cues. These modifications—primarily acetylation, methylation, and phosphorylation—impact chromatin accessibility and consequently the expression of hormone-related genes. A growing body of research highlights the intricate crosstalk between histone modifications and hormonal pathways in plants, positioning histone dynamics as critical regulators of hormonal biosynthesis, signaling, and response coordination during key stages such as germination, ripening, and stress adaptation [14,15].

Histone acetylation, catalyzed by histone acetyltransferases (HATs), typically correlates with transcriptional activation by loosening chromatin structure, whereas histone deacetylases (HDACs) remove acetyl groups and restore repression [14]. Histone methylation is more precise: tri-methylation of H3K4 and H3K36 is associated with active transcription, while H3K27me3 and H3K9me2 generally mediate gene silencing [16]. These marks are dynamically altered in response to internal cues such as hormone levels or external stressors like salinity and pathogens [17].

During seed germination, the repression of maturation-phase genes and activation of vegetative genes is controlled by Polycomb Repressive Complexes PRC1 and PRC2 through H3K27me3-mediated silencing. These complexes are recruited by transcriptional repressors such as VAL1 and VAL2 to suppress LAFL genes (*LEC1*, *ABI3*, *FUS3*, *LEC2*)—central to ABA signaling—thereby promoting GA-mediated germination [15,18]. PICKLE (PKL), a chromatin remodeling factor, also plays a role by antagonizing LAFL activity, further facilitating hormonal transition [19].

In *Arabidopsis*, histone acetylation is dynamically modulated during seedling development to control the expression of GA biosynthetic genes, whereas HDACs such as *HDA6* and *HDA19* repress ABA-responsive genes during seedling growth [14,15,18]. Likewise, in tomatoes, *HDA3* and *HDA1* repress ripening by inhibiting ethylene biosynthesis and perception genes until developmental cues remove repression via histone acetylation [20,21].

Epigenetic control over hormone biosynthesis genes is well established. In climacteric fruits such as tomatoes and bananas, H3K27me3 represses key ripening genes including ACO and ACS, which encode enzymes for ethylene biosynthesis. Demethylation of H3K27me3 at these loci coincides with ethylene bursts and the onset of ripening [20,21]. Additionally, ABA biosynthesis genes (NCED) show activation through increased H3K4me3 levels, reflecting permissive chromatin under stress or ripening cues [16,21].

In sugar signaling contexts, which integrate with hormonal pathways, chromatin remodelers and histone acetylation facilitate the activation of hormone-sensitive genes. HXK1, a glucose sensor, recruits histone-modifying complexes to modulate auxin and cytokinin signaling genes under sugar-rich conditions [22].

Under abiotic stresses like salinity or drought, histone modifications provide plants with transcriptional plasticity. In response to salt stress, increased H3 acetylation (e.g., H3K9ac, H3K27ac) at promoters of stress-responsive genes correlates with ABA accumulation and activation of signaling pathways [16]. Similarly, methylation of H3K4 at genes involved in JA and SA signaling has been documented during pathogen response, reflecting pathogen-triggered immunity (PTI) activation [17].

Moreover, specific histone methyltransferases (HMTs) and demethylases (e.g., JMJ family proteins) respond to hormonal signals and ROS cues, modulating the histone code to control stress hormone pathways [16,18]. PRC2 and HDA19 repression of ABA-inducible transcription factors under non-stress conditions ensure homeostasis, while their derepression during stress allows rapid hormonal responses [14,16].

Histone modifications frequently work in tandem with DNA 14 and miRNAs, especially in developmental reprogramming events. For example, sugar- and hormone-responsive genes often exhibit coordinated changes in DNA methylation and histone acetylation/methylation, forming a multi-layered regulatory landscape [22]. In seed germination, ROS-induced chromatin remodeling leads to increased H3 acetylation and reduced H3K27me3, thereby promoting GA biosynthesis and ABA degradation [18].

### 2.3. Small RNAs in Hormonal Pathways: Post-Transcriptional Regulation of Plant Development and Immunity

Small RNAs (sRNAs), including microRNAs (miRNAs) and small interfering RNAs (siRNAs), are critical components of gene regulatory networks in plants. By guiding post-transcriptional gene silencing, they fine-tune gene expression with spatial and temporal precision. In recent years, sRNAs have been linked to the regulation of hormonal biosynthesis and signaling pathways, positioning them as central players in the coordination of growth, development, and stress responses [8].

Plant miRNAs typically function by guiding the RNA-induced silencing complex (RISC) to complementary mRNA targets, resulting in transcript cleavage or translational repression. Many hormone-responsive genes—including transcription factors and receptors—are direct targets of miRNAs [23,24]. For example, the miR160 and miR167 families regulate auxin signaling by targeting ARF10/16/17 and ARF6/8, respectively, which encode auxin response factors [23]. Similarly, miR159 and miR319 modulate GA and JA signaling through MYB and TCP transcription factors [25,26].

In the ABA pathway, miR168 regulates ARGONAUTE1 (AGO1), a key effector of the miRNA pathway itself, forming a feedback loop between sRNA biogenesis and ABA sensitivity [27]. miR393, which targets auxin receptors TIR1 and AFB2, is induced by abiotic stress and serves as a rapid switch that attenuates auxin signaling, thereby enhancing stress tolerance [23,26].

sRNAs play essential roles in regulating hormone-driven developmental transitions. The miR156–SPL module, for instance, is involved in the juvenile-to-adult phase transition, flowering time, and inflorescence development through interactions with GA and ABA pathways [23,25]. In rice, miR5488 and miR399 are implicated in thermosensitive male sterility, partly through influencing lignin biosynthesis and flavonoid metabolism—processes tightly linked to hormone action [25].

During seed germination, a finely tuned interplay between ABA and GA determines dormancy and activation. Small RNAs modulate this balance by targeting key hormone metabolism genes. For example, miR159-mediated repression of MYB33/MYB101 transcription factors reduces ABA sensitivity, promoting germination [27].

sRNAs also act as mediators of hormone-regulated defense responses. The miR393–TIR1 module enhances SA signaling and suppresses auxin signaling, creating a hormonal environment favoring defense overgrowth [24,26]. Viral infections further trigger sRNA expression shifts that impact hormonal crosstalk; for instance, induction of miR171 and miR168 affects ABA, auxin, and JA signaling, which viruses may exploit to enhance replication and systemic spread [24,28].

In *Arabidopsis*, SA upregulates miR472, which suppresses resistance (R) genes, fine-tuning the balance between immune activation and growth [26]. JA- and ET-pathways are also targeted by sRNAs such as miR319, adjusting defense output based on the invading pathogen [29].

Stress exposure can lead to transgenerational reprogramming of sRNA expression, impacting hormone-related gene expression in offspring. In durum wheat, water-deficit stress experienced by parental plants triggered changes in miRNAs regulating ABA and cytokinin pathways, enhancing progeny resilience [6].

These effects align with stress-induced epigenetic memory, where sRNAs help stabilize hormone-related transcription patterns across generations, offering potential targets for breeding stress-resilient crops [6,27].

## 3. Hormonal Balance in Progeny: Epigenetic Effects on Fitness

The capacity of plants to transmit environmental experience to their progeny has fundamentally reshaped our understanding of plant adaptation and fitness. Far beyond traditional Mendelian inheritance, transgenerational epigenetic mechanisms—most notably DNA methylation, histone modifications, and small RNAs—can modulate hormonal balance in offspring, influencing developmental trajectories and ecological success.

Plants subjected to abiotic stressors such as drought or salinity often exhibit persistent changes in hormonal regulation, which are partially inherited by their descendants. This transmission of ”stress memory” is frequently mediated by epigenetic modifications that alter the expression of hormone biosynthesis or signaling genes [7]. For example, enhanced ABA signaling, a hallmark of stress priming, can persist in progeny due to maintained chromatin states or stable DNA methylation patterns established during parental stress exposure [12].

The maintenance of such epigenetic marks during reproductive development—especially in germline precursor cells—enables the inheritance of primed states. These modifications modulate hormone homeostasis in developing seeds, influencing dormancy, germination, and early growth responses to environmental conditions.

Maternal plants exposed to stress conditions often alter the hormonal content of seeds, either through direct provisioning or epigenetic programming of hormone metabolism. For example, altered auxin or gibberellin levels in seeds derived from stressed parents have been associated with delayed germination or enhanced stress tolerance [30].

This effect can be further shaped by environmental interactions, such as beneficial symbioses. Arbuscular mycorrhizal (AM) symbiosis can alleviate drought stress in *Taraxacum brevicorniculatum* but also can trigger transgenerational phenotypic plasticity, including changes in root architecture and resource-use traits in progeny [31]. These changes were closely linked to hormonal pathways and suggest that epigenetic mechanisms control the balance between stress-responsiveness and growth-promoting hormones across generations.

Although epigenetic memory can confer enhanced resilience, it can also result in hormonal imbalances that compromise growth under favorable conditions. For instance, a primed ABA state may increase survival during drought but impair seedling vigor if water is abundant [32]. Thus, the persistence of epigenetic marks regulating hormonal crosstalk must be finely tuned to environmental predictability.

Importantly, fitness advantages conveyed by epigenetic-hormonal modifications tend to be most pronounced during early developmental stages—especially in seedling establishment and root growth—where hormonal regulation plays a dominant role [31]. In perennial plants, partial maintenance of the epigenetic landscape across seasons may contribute to long-term adaptations, such as adjusted hormone sensitivity and seasonal phenology [7].

Plants exhibit dynamic control over the inheritance of epigenetic marks. While some marks are faithfully transmitted through meiosis, others are reset during gametogenesis, a process influenced by both hormonal signals and developmental context [12]. This controlled resetting allows flexibility: it preserves beneficial adaptations while avoiding maladaptive stress legacies.

Recent studies suggest that selective retention of epigenetic states regulating hormone biosynthesis and perception allows for a refined strategy—transmitting only those hormonal cues that increase progeny fitness under anticipated environmental scenarios [30,32].

### 3.1. Auxin and Root–Shoot Allocation: Epigenetic Regulation of Growth Plasticity

Plant developmental plasticity, especially in root–shoot allocation, is a key adaptive mechanism for optimizing resource acquisition in dynamic environments. Central to this plasticity is the plant hormone auxin, which orchestrates differential growth patterns and organ development through tightly regulated spatial gradients. Emerging evidence has revealed that epigenetic regulation—including chromatin remodeling, histone modifications, DNA methylation, and non-coding RNAs—modulates auxin biosynthesis, transport, and signaling to fine-tune root–shoot balance under varying environmental and physiological contexts.

Auxin distribution is critical in shaping root system architecture (RSA) and shoot development. Local auxin maxima, particularly in the root apical meristem, govern lateral root initiation and elongation, while systemic auxin gradients integrate signals from shoot-derived photosynthates, nutrient availability, and stress conditions [33]. Mutations in YUCCA, TAA1, and PIN gene families, which regulate auxin biosynthesis and transport, consistently disrupt root development and alter root-to-shoot ratios in both model and crop species such as rice, maize, and pea [33].

Moreover, auxin interacts with other hormones such as cytokinins, ABA, and gibberellins to modulate organ growth in a context-dependent manner. For example, heat stress triggers root–shoot signaling involving auxin and ABA, with the auxin pool in roots contributing to meristem maintenance and stress adaptation [34]. Crosstalk with cytokinin and ethylene further refines RSA to optimize growth under nutrient stress or thermal extremes.

Environmental cues frequently alter auxin-related gene expression through epigenetic modifications. Under phosphate and sulfur starvation, DNA methylation patterns in YUCCA and PIN promoters are reorganized to induce local auxin biosynthesis and redirect root growth to favorable zones [23]. Simultaneously, histone acetylation and H3K4me3 marks activate auxin-responsive genes in the root tip, enhancing adaptive root elongation and branching [34].

The Target of Rapamycin (TOR) kinase pathway integrates nutrient availability and sugar signaling with auxin responses. Under sulfur deficiency, autophagy is upregulated in shoots while root TOR activity is preserved, facilitating carbon allocation and supporting auxin-induced meristem activity [23]. This balance is critical for root growth under nutrient stress and directly links energy sensing with hormone-regulated developmental plasticity.

The TOR–auxin axis also intersects with chromatin-level regulation. TOR activity modulates the expression of WUSCHEL and E2Fa in a tissue-specific manner, thereby controlling meristem size and activity in a way that responds both to internal metabolic status and external environmental signals [23].

Sulfate limitation, for instance, downregulates TOR activity in shoots but maintains it in roots via glucose-TOR signaling, preserving auxin-mediated root apical meristem activity. This organ-specific control enables enhanced root growth at the expense of shoots, a classical example of an increased root-to-shoot ratio during stress [23].

Chromatin remodeling under thermal and salt stress also regulates auxin responses by modulating access to auxin transporters and signaling genes. This epigenetic plasticity contributes to maintaining functional RSA under hostile conditions, such as salinity and high temperature, where meristem function must be preserved despite compromised cellular metabolism [34,35].

Root–shoot plasticity is not merely a physiological adjustment but an evolved developmental strategy. Plants dynamically regulate root branching, elongation, and shoot architecture to optimize carbon–nutrient balance, especially under stress or soil constraints [36,37]. Genetic diversity in wheat, for instance, has revealed alleles contributing to enhanced plasticity under sodic and acidic soils—traits associated with epigenetically regulated auxin and ABA pathways [37].

Modern breeding strategies increasingly target these epigenetically controlled traits to enhance resilience in cereal crops. Epigenomic profiling, coupled with high-resolution phenotyping of RSA traits, is enabling the identification of candidate regulators and epialleles that confer beneficial root–shoot allocation patterns [35].

### 3.2. Abscisic Acid and Stress Tolerance: Epigenetic Memory of Drought Response

Abscisic acid is a central hormonal mediator of plant responses to drought, functioning as both a rapid-response signal and a key regulator of long-term acclimation. Recent research has revealed that epigenetic mechanisms—including DNA methylation, histone modifications, and chromatin remodeling—play essential roles in establishing and maintaining stress memory, enabling plants to mount enhanced responses to recurrent drought episodes. These insights not only deepen our understanding of plant resilience but also offer promising avenues for crop improvement in the face of climate change.

Under drought stress, increased ABA accumulation promotes stomatal closure, osmotic adjustment, and activation of ABA-responsive transcription factors (TFs) such as AREB/ABF and DREB families, which regulate gene networks related to dehydration tolerance [38]. Many of these ABA-inducible genes exhibit transcriptional memory, characterized by sustained expression or faster re-induction upon subsequent drought events.

Stress-induced changes in chromatin architecture—particularly DNA methylation and histone modifications—are essential for encoding drought memory. Repeated drought exposure leads to hypomethylation of drought-responsive gene promoters (e.g., RD29A, DREB2A) and enrichment of permissive histone marks such as H3K4me3 and H3K9ac, which maintain these loci in a “poised” transcriptional state [38,39].

In *Arabidopsis*, memory genes remain in a transcriptionally competent state during recovery phases between drought episodes, with paused RNA Polymerase II ready to resume transcription rapidly during renewed stress [39]. The stability of these marks during mitotic divisions enables somatic stress memory, ensuring that already differentiated tissues retain enhanced drought responsiveness.

Beyond somatic memory, drought-induced epigenetic changes can be transmitted to progeny, resulting in transgenerational stress memory. This has been observed in several crop species, including citrus, where grafting with buds from drought-experienced plants improved stress tolerance and photosynthetic efficiency in new individuals [40]. Such effects are often genotype-dependent, with specific rootstock–scion combinations better preserving epigenetic imprints related to ABA regulation.

Studies in wheat, rice, and poplar have shown that specific DNA methylation patterns and histone modifications established during drought can persist in germline cells, altering ABA-related gene expression in the next generation [25,41]. This heritability may occur via partial escape from meiotic resetting, allowing stress memory to bypass epigenetic reprogramming checkpoints [39,42].

Small RNAs, particularly siRNAs and miRNAs, modulate ABA signaling through post-transcriptional silencing of target genes involved in hormone biosynthesis and signal transduction. Moreover, the RNA-directed DNA methylation (RdDM) pathway reinforces transcriptional silencing and can contribute to stable memory marks [43]. These RNA-mediated pathways are integral in reinforcing chromatin states associated with ABA-responsive loci during stress priming and recovery.

Harnessing epigenetic memory of ABA-mediated drought tolerance offers great potential for sustainable agriculture. Priming strategies, such as drought pre-treatment or seed priming, induce beneficial epigenetic states that can be retained across developmental stages or even inherited, thereby enhancing tolerance without genetic modification [44]. The combination of natural variation, epigenome editing, and epigenetic breeding is an emerging frontier to exploit ABA-associated memory for climate-resilient crop varieties.

### 3.3. Gibberellins and Growth Plasticity: Epigenetic Regulation of Developmental Timing

Gibberellins are hormones that regulate a wide range of developmental processes, including seed germination, stem elongation, and flowering time. The regulation of GA signaling is particularly important for plant growth plasticity under stress, as it allows plants to adjust their growth patterns to optimize survival.

Epigenetic regulation of GA signaling is mediated by DNA methylation and histone modifications. DNA methylation of genes involved in GA biosynthesis or signaling, such as GA20ox, can influence seed germination and stem elongation under stress conditions [45]. For example, in response to heat stress, plants may adjust the timing of germination or elongate stems to avoid adverse conditions, with these changes being regulated by epigenetic modifications in GA-related genes.

In progeny, epigenetic modifications in GA pathways can have long-term effects on developmental timing. Transgenerational inheritance of epigenetic marks that regulate GA signaling can lead to offspring with altered growth patterns, such as delayed flowering or increased stem elongation, which may confer survival advantages under fluctuating environmental conditions [46]. This form of epigenetic regulation of GA signaling provides an example of how plant development can be tailored to specific environmental challenges, ensuring that offspring are better equipped to cope with future stressors.

### 3.4. Jasmonic Acid and Defense Responses: Epigenetic Regulation of Herbivore Resistance

Jasmonic acid is a key hormone involved in plant defense responses, particularly against herbivores and pathogens. Epigenetic regulation of JA signaling pathways has important implications for the defense responses of progeny, as it enables plants to “remember” prior herbivore attacks and enhance their defense mechanisms in subsequent generations.

DNA methylation and histone modifications regulate the expression of JA-responsive genes, which control defense-related proteins such as proteinase inhibitors and defensins. Epigenetic modifications at the promoters of these genes can enhance their expression in response to herbivory [47]. For example, DNA methylation of genes involved in the biosynthesis of JA or the expression of JA receptors can result in increased resistance to herbivores and pathogens. These epigenetic marks can be inherited, allowing progeny to mount a stronger defense response without the need for an initial herbivore attack.

Small RNA regulation also plays a role in the inheritance of JA-related defense responses. Small RNAs, particularly siRNAs, can target and silence genes involved in JA biosynthesis or receptor expression, providing a mechanism for transgenerational memory of herbivore attacks [48]. By inheriting these small RNA-mediated marks, progeny is able to respond more effectively to herbivore pressure, ensuring increased fitness in environments where biotic stresses are common.

## 4. Transgenerational Epigenetic Inheritance of Hormonal Balance: Implications for Crop Improvement

The inheritance of epigenetic modifications in hormonal pathways has significant implications for crop breeding and the development of stress-resilient varieties. Through epigenetic imprinting, plants can pass down enhanced stress responses to their progeny, enabling them to cope with similar environmental conditions in the future. This transgenerational adaptation provides a form of “stress priming”, where progeny is better prepared to face environmental challenges such as drought, salinity, and pathogen attacks.

Understanding how epigenetic modifications influence hormonal pathways allows for the potential manipulation of these pathways to enhance crop resilience. By selecting crops with favorable epigenetic marks that promote stress tolerance, we can improve yield stability under adverse environmental conditions. Additionally, the use of epigenetic priming to activate stress-responsive pathways in crops can lead to enhanced productivity, particularly in regions affected by climate change [47].

### Potential for Crop Improvement Through Epigenetic Priming

The ability to manipulate epigenetic pathways to improve crop resilience has significant implications for agriculture. Epigenetic priming involves the pre-conditioning of plants through environmental cues or controlled stress exposure, leading to beneficial epigenetic modifications that enhance stress tolerance in future generations. This process can be particularly useful in crop breeding, as it offers a way to enhance resilience without altering the plant’s genetic makeup.

Recent studies have demonstrated that epigenetic priming can trigger long-lasting modifications in chromatin and DNA methylation patterns, particularly in genes involved in ABA, JA, and auxin pathways. For instance, in *Oryza sativa*, mild drought stress led to hypomethylation of promoters of ABA biosynthesis genes (e.g., *OsNCED*), and these changes persisted across at least two generations, resulting in enhanced drought avoidance and increased water use efficiency [49]. Similarly, in *Zea mays*, transgenerational memory of heat and drought stress was linked to differential expression of small RNAs targeting stress-responsive transcription factors, contributing to improved seedling vigor and root development in the progeny.

In *Zea mays* (maize), stress priming under controlled heat and drought conditions resulted in stable transgenerational upregulation of miRNAs such as miR168 and miR399, which regulate genes involved in hormone signaling and oxidative stress detoxification. These miRNAs were retained in seedlings derived from stressed parents, contributing to enhanced germination, seedling vigor, and stress preparedness, even in the absence of direct stress exposure [6].

Similarly, in *Brassica napus*, salt-primed parental plants produced seeds with altered expression of histone methyltransferases (e.g., *SUVH4*), resulting in differential H3K9me2 marks on stress-inducible genes in progeny. These chromatin changes correlated with increased expression of ion transporters like *NHX1*, promoting salt exclusion and maintaining ion homeostasis under salinity.

Moreover, priming with beneficial microbes, such as plant growth-promoting rhizobacteria (PGPR) or arbuscular mycorrhizal fungi (AMF), has been shown to trigger epigenetic changes in stress-related hormone pathways. For example, AMF-inoculated taraxacum plants exposed to drought exhibited not only improved stress tolerance in the parental generation but also transgenerational shifts in auxin-to-ABA balance and modifications in root architecture in their offspring, mediated by changes in DNA methylation [50].

By exposing plants to mild drought conditions, it is possible to induce epigenetic modifications in stress-responsive genes such as those involved in ABA biosynthesis. These modifications can be passed down to progeny, resulting in improved drought tolerance in future generations. Additionally, epigenetic priming can enhance the plant’s ability to respond more rapidly and effectively to subsequent drought events, reducing water usage and improving crop productivity in water-limited regions.

Similarly, exposing plants to mild pathogen stress can induce epigenetic modifications in genes involved in the JA signaling pathway, which is crucial for plant defense responses. These modifications can be inherited by progeny, providing them with enhanced resistance to pathogens such as fungi, bacteria, or viruses. Epigenetic priming for pathogen resistance can improve crop health and yield, reducing the need for chemical pesticides and fostering more sustainable agricultural practices.

From a breeding perspective, epigenetic priming provides a complementary tool to traditional genetic selection. Marker-assisted selection targeting stable epialleles—heritable epigenetic variants affecting traits such as root architecture, flowering time, or pathogen resistance—is gaining momentum. Moreover, emerging technologies such as CRISPR/dCas9-based epigenome editing now allow targeted activation or silencing of stress-regulatory genes without changing the underlying DNA sequence. High-throughput epigenomic profiling platforms are also being integrated into pre-breeding programs to identify elite lines with desirable epigenetic marks [7,12].

In parallel, advances in epigenome editing technologies, such as CRISPR/dCas9 fused to DNA methyltransferases (e.g., DNMT3A) or demethylases (e.g., TET1), now enable targeted modifications of DNA methylation patterns at specific loci without altering nucleotide sequences [7]. These tools have the potential to precisely modulate gene expression, for instance by demethylating promoters of ABA or JA biosynthesis genes to enhance drought or pathogen resistance.

High-throughput epigenomic profiling platforms, including whole-genome bisulfite sequencing (WGBS) and chromatin immunoprecipitation sequencing (ChIP-seq), are increasingly incorporated into pre-breeding pipelines to map variation in methylation and histone modification landscapes. This facilitates the screening of natural epigenetic diversity across landraces, wild relatives, and cultivated varieties to identify epigenotypes with superior stress responses under real-world field conditions [12].

These advances offer a strategic framework for integrating epigenetic knowledge into breeding programs, enabling the development of crop varieties that are not only genetically robust but also epigenetically primed for resilience, particularly in the face of climate change. This epigenome-informed breeding approach provides a sustainable and innovative alternative to conventional improvement strategies, especially for traits where gene expression plasticity plays a central role [7].

## 5. Challenges and Future Directions

While the potential for epigenetic priming in crop improvement is promising, there are several challenges that need to be addressed. One key issue is understanding the stability and reversibility of epigenetic marks. Although some modifications are stable and can be passed on to progeny, others may be reversible or reset under favorable conditions. Future research should focus on identifying which epigenetic marks are most stable and beneficial for long-term stress tolerance and how these marks can be reliably inherited in crop species.

Another challenge is the genetic diversity of crops. While epigenetic priming offers a mechanism for enhancing stress tolerance, it must be integrated with traditional breeding practices to ensure that genetic diversity is maintained. Over-reliance on epigenetic priming could limit the adaptive potential of crops if genetic variation is reduced.

Precision breeding techniques, such as CRISPR/Cas9, offer the potential to directly edit epigenetic marks associated with stress tolerance. However, the long-term impacts of such interventions on crop performance, yield, and biodiversity must be carefully considered before widespread implementation.

## 6. Conclusions

The epigenetic regulation of hormonal pathways plays a crucial role in shaping plant responses to environmental stresses. These modifications not only influence growth and stress tolerance but also provide progeny with an adaptive advantage through transgenerational memory. By understanding the mechanisms that regulate these hormonal pathways, we can develop strategies to enhance crop resilience and productivity, particularly in the face of climate change. Future research will be essential to unravel the complexities of epigenetic inheritance and its role in improving plant resilience through epigenetic priming.

## Abbreviations

The following abbreviations are used in this manuscript:

ABA	Abscisic acid
AM	Arbuscular mycorrhizal
ACS	1-aminocyclopropane-1-carboxylate synthase
ACO	1-aminocyclopropane-1-carboxylate oxidase
DME	DEMETER DNA glycosylase
DRM	Domains rearranged methyltransferase
GA	Gibberellin
GUS	β-glucuronidase
HAT	Histone acetyltransferase
HDAC	Histone deacetylase
HMT	Histone methyltransferase
JA	Jasmonic acid
LAFL	LEC1, ABI3, FUS3, and LEC2 gene network (seed maturation regulators)
miRNA	MicroRNA
PRC1/PRC2	Polycomb repressive complex 1/2
PTI	Pathogen-triggered immunity
RdDM	RNA-directed DNA methylation
RISC	RNA-induced silencing complex
RNA Pol II	RNA polymerase II
ROS	Reactive oxygen species
RSA	Root system architecture
SA	Salicylic acid
siRNA	Small interfering RNA
sRNA	Small RNA
TOR	Target of rapamycin
